# The role of gender in a smoking cessation intervention: a cluster randomized clinical trial

**DOI:** 10.1186/1471-2458-11-369

**Published:** 2011-05-23

**Authors:** Diana Puente, Carmen Cabezas, Teresa Rodriguez-Blanco, Carmen Fernández-Alonso, Tránsito Cebrian, Miguel Torrecilla, Lourdes Clemente, Carlos Martín

**Affiliations:** 1 IDIAP J Gol, Av. Gran Via de les Corts Catalanes 587, 08007-Barcelona, Spain; 2 Departament de Salut, Generalitat de Catalunya, Roc Boronat 81-95, 08005-Barcelona, Spain; 3 Servicio de Coordinación Sociosanitaria, Consejería de Sanidad, Paseo de Zorrilla 1, 47007-Valladolid, Spain; 4 Distrito Sanitario Aljarafe. Servicio Andaluz de Salud. Junta de Andalucía, Av. de las Américas s/n, 41927-De Mairena del Aljarafe, Sevilla, Spain; 5 Centro de Salud San Juan, Valencia 32, 37005-Salamanca, Spain; 6 Centro de Salud Santo Grial, San Jorge 38, 22003-Huesca, Spain; 7 Centre Atenció Primària Passeig de Sant Joan, Passeig de Sant Joan 20, 08010-Barcelona, Spain

**Keywords:** gender, smoking cessation, primary health care, clinical trials

## Abstract

**Background:**

The prevalence of smoking in Spain is high in both men and women. The aim of our study was to evaluate the role of gender in the effectiveness of a specific smoking cessation intervention conducted in Spain.

**Methods:**

This study was a secondary analysis of a cluster randomized clinical trial in which the randomization unit was the Basic Care Unit (family physician and nurse who care for the same group of patients). The intervention consisted of a six-month period of implementing the recommendations of a Clinical Practice Guideline. A total of 2,937 current smokers at 82 Primary Care Centers in 13 different regions of Spain were included (2003-2005). The success rate was measured by a six-month continued abstinence rate at the one-year follow-up. A logistic mixed-effects regression model, taking Basic Care Units as random-effect parameter, was performed in order to analyze gender as a predictor of smoking cessation.

**Results:**

At the one-year follow-up, the six-month continuous abstinence quit rate was 9.4% in men and 8.5% in women (p = 0.400). The logistic mixed-effects regression model showed that women did not have a higher odds of being an ex-smoker than men after the analysis was adjusted for confounders (OR adjusted = 0.9, 95% CI = 0.7-1.2).

**Conclusions:**

Gender does not appear to be a predictor of smoking cessation at the one-year follow-up in individuals presenting at Primary Care Centers.

**ClinicalTrials.gov Identifier:**

NCT00125905.

## Background

Smoking has devastating effects on health [1] and is the first avoidable cause of mortality in developed countries [2]. In Spain, there were 53,155 deaths attributable to tobacco use in 2006 (14.7% of all deaths occurred in individuals > = 35 years), 47,174 in men and 5,981 in women [3].

Over the last decade, global tobacco consumption has decreased progressively in Spain; however, tobacco consumption remains high. According to data from 2006, 21.5% of women and 31.6% of men older than 16 were current smokers in Spain [4]. Specifically, until the 1970s, the prevalence of smoking in women was lower than 5%. Tobacco consumption in women has since increased, reaching peak levels around 1995 and 2001, and falling slightly in the following decade. In men, tobacco consumption began to decrease after the mid 1970s [5].

In light of this prevalence of smoking in both genders, it is necessary to implement specific intervention programs for smoking cessation in Primary Care Units. Until recently, tobacco control initiatives have not differentiated between the two gender groups; however, attitudes, perceptions and behaviors related to tobacco consumption patterns and cessation can differ between men and women [5-8]. Therefore, knowledge of the mechanisms by which gender influences abstinence could provide targets for intervention [9].

Interventions aimed at smoking cessation are sometimes based on models such as the Transtheoretical Change Model [10]. According to this model, smoking cessation is a process of movement through motivational stages of change, from no intention to quit to maintaining smoking cessation [11]. From the point of view of the Transtheoretical Change Model, many factors, such as attitude, social influence, self-efficacy, employment, age, marital status and gender, among others, could be key factors in determining behavior and change of stage.

The main purpose of this project was to evaluate gender as a predictor of smoking cessation in a smoking cessation intervention ("Intervención Sistemática sobre Tabaquismo en Atención Primaria de Salud- ISTAPS") using motivational interviews and the stages of change model [12]. Additionally, we assessed potential differences in the pattern of tobacco use by gender at baseline and at one year after inclusion in the study.

## Methods

This article is based on the ISTAPS study.

### ISTAPS study

The ISTAPS Study evaluated the effectiveness of a stepped smoking cessation intervention based on Prochaska and DiClemente's Transtheoretical Change Model [10]. This study was a cluster randomized clinical trial in which the randomization unit was the "Basic Care Unit" (BCU). A BCU comprises a family physician and a nurse who are in charge of the care for a given population of patients. Subjects were recruited from 82 Primary Health Care Centers in 13 regions of Spain.

The intervention consisted of a six-month period of structured and systematic recommendations implemented from a Clinical Practice Guideline based on the "cessation-induction trials" model proposed by Hughes et al [13], including: motivational counseling for smokers at the precontemplation/contemplation stage; brief intervention for smokers in preparation/action who did not want help; intensive intervention with drug therapy for smokers in preparation/action who wanted help; and reinforcing intervention in the maintenance stage. The guidelines that served as the basis of the intervention had been published recently [14]. The control group received usual care (standard care), including smoking cessation counseling offered in primary care to patients with diseases related to tobacco consumption. This is enterely free of charge, as the Spanish National Health Service (NHS) provides universal medical coverage and is financed essentially by general taxes. The usual care could also include drug therapies, although these treatments were not covered by the National Health Service.

Information related to process of change, decisional balance, self-efficacy and quality of life was collected through telephone interviews. Information related to socio-demographics, lifestyles and pattern of tobacco consumption was collected through personal interviews. Finally, clinical information was collected from the subjects' medical records. Informed consent to participate was obtained from all study subjects.

The follow-up period was two years from the start of the intervention. For this project, we have restricted our analysis to the first year of follow-up.

Definitive approval from the Clinical Ethics Committee of the IDIAP Jordi Gol has been granted for the ISTAPS project (P02/13).

Detailed information about the ISTAPS project has been published previously [12].

### Study population

Our population was based on 2,937 subjects between 14 and 75 years of age from the ISTAPS study, recruited from 2003 to 2005. Subjects were individuals who presented at Primary Health Care Centers for any reason and answered "yes" to the question: "Do you smoke cigarettes?". Of 2,937 subjects, 2,827 with valid information were selected for the analysis.

### Data collection

Baseline information on socio-demographics, lifestyles, tobacco use, stages of change, nicotine dependence (Fagerström test scores) [15], motivation to quit (Richmond test scores) [16], confidence in quitting, importance of quitting and readiness to quit as defined by Rollnick et al [17] was collected through personal questionnaires administered by the doctor or nurse in the Primary Care Center.

Age in years, age at smoking initation, alcohol consumption, number of cigarettes per day, Fagerström and Richmond test scores, importance of quitting smoking, confidence in quitting smoking and readiness to quit smoking, measured using a scale from 0 (lowest) to 10 (highest), were analyzed as continuous variables. The measure of alcohol consumption was based on the standard unit of alcohol (1 unit = 10 gr). Alcohol consumption was measured as standard units of alcohol per week.

It is interesting to mention the Richmond test. This test is used very widely in Spain and was described by RL. Richmond in 1993. The test measures the smoker's motivation to quit smoking.

Social class was categorized according to the British Registrar General's Social Classification (2000) and codified into two categories (I-II: professionals, managerial and technical occupations/III-V: non-manual skilled and manual skilled occupations, partly skilled and unskilled occupations) [18]. Physical exercise (leisure time) was also classified into two categories, one for subjects who exercised at least once a week versus those who did not exercise at all. Categorical variables also included: group allocation (control/intervention), smoker partner (yes/no), smoker friends (yes/no), stage of change (pre-contemplation: the individual has no intention to change behavior in the next six months; contemplation: the individual recognizes the benefits of changing and is considering taking action in the next six months; preparation: the individual has decided to make a change in the next 30 days; action: the individual is actively engaged in changing their behavior), number of previous smoking cessation attempts lasting at least 24 hours (from 0 to 5), reduction in the number of cigarettes consumed over last month (yes/no) and presence of tobacco related disease (yes/no). The maintenance and relapse stages were not considered since the study focused on active smokers. A few subjects were in the action stage at baseline because they gave up smoking between the recruitment day and the interview day.

Some study subjects (21% of men and 19.4% of women) received treatment during the study period. Treatment was defined as both drug therapy and supplemental treatments, such as anti-anxiety agents, candies, regular gums and books. Drug therapy included medications such as bupropion and nicotine substitutes (gums and patches) that were part of the intervention. Since the intervention is considered as an indivisible whole, the impact of treatment on the primary outcome cannot be known. The control group, which included brief advice for smokers, also used these drug therapies in some cases, but not in the same protocolized way as in the intervention group. For this reason, a greater use of pharmacological treatment in the intervention than the control group is normal and desirable. Thus, treatment could not be included as a separate variable because it was assessed basically as a part of the intervention or usual care.

The dichotomous outcome variable was selected, comparing those patients who had been ex-smokers for at least six months of continuous abstinence (self-reported) versus those who were current smokers at the end of study period (one year). Subjects who were former smokers for less than six months were considered smokers Initially all subjects were asked to confirm their self-reported abstinence on the expired air carbon monoxide test. Smoking abstinence was considered to be confirmed at a carbon monoxide level lower than 10 ppm. Only 12% of former smokers could be confirmed biochemically.

### Data analysis

Differences in socio-demographics, lifestyles and tobacco characteristics at baseline and at one-year follow-up were assessed in men versus women using the chi-square and Student's t-test or Mann-Whitney U as appropriate.

Linear regression models were used to assess the independent predictor variables on number of cigarettes consumed last month in men, importance, confidence and readiness to quit at the one-year follow-up in both genders, adjusted for baseline measurement. The standard errors were adjusted for the cluster effect of the BCU. Because significant variability among BCUs was found in the number of cigarettes consumed last month in women, a linear mixed-effects model was performed to assess the independent predictors on number of cigarettes consumed last month. These analyses were stratified by gender.

The initial individual variables considered were study group, age, social class, alcohol intake, physical exercise, number of cigarettes consumed last month, Fagerström test scores, Richmond test scores, smoker partner, smoking reduction over last month, importance of quitting smoking, confidence in quitting smoking, readiness to quit smoking and tobacco-related disease.

All subjects were included in the groups to which they were randomized and an intention-to-treat analysis was performed. Additionally, subjects lost to follow-up were considered unsuccessful in their attempt to quit smoking and therefore current smokers, which is the standard approach in smoking cessation intervention studies [19].

A logistic mixed-effects model was performed to assess gender as a predictor of continuous smoking abstinence, accounting for clustering at the level of BCU [20]. We used the binomial logit link with full maximum likelihood method of estimation via the adaptative Gaussian quadrature with fifty integration points.

We modeled individuals (level-1 units) as nested within BCUs (level-2 units).

Initially we examined whether there was significant variability among BCUs in the likelihood of smoking cessation fitting an unconditional model with no predictors at either level. As significant variation existed between BCUs, we built a conditional model adding level-1 individual fixed effects. We did not consider contextual variables at the BCU level.

The initial regression model was adjusted for gender, study group, age, social class, alcohol intake, physical exercise, age at smoking initation, number of cigarettes consumed last month, Fagerström test scores, Richmond test scores, smoker partner, smoker friends, stage of change, number of previous smoking cessation attempts lasting at least 24 hours, reduction of number of cigarettes consumed over the last month, importance of quitting smoking, confidence in quitting smoking, readiness to quit smoking and tobacco-related disease. The method recommended by Raudenbaush & Bryck (2002) [21] was followed to select a subset of covariates to include in the final regression model. The authors checked for confounders and multi-colinearity among the independent variables. Homogeneity of the group effect over men and women was verified, and therefore this interaction was not included in the model.

The alternative models were compared using the partial likelihood ratio test and Akaike's information criterion (AIC) to determine which model provided the best fit for the data. All results were expressed with their 95% confidence intervals (CIs) Statistical significance was set at p < 0.05 (two-tailed).

Data were managed using the ACCESS databases, and statistical analysis was performed using the SPSS statistical package for Windows, version 13.0 (SPSS Inc., Chicago IL) and Stata/SE version 11.1 statistical package (Stata Corp).

## Results

**Figure 1:**
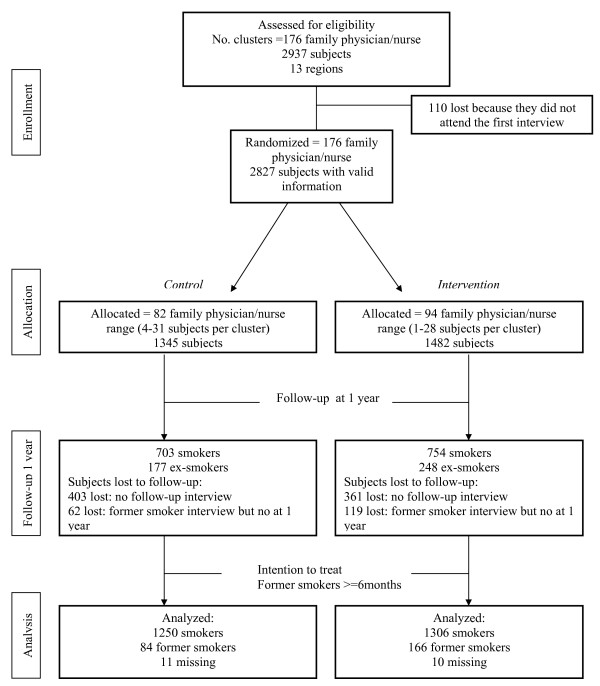
**Flowchart of the ISTAPS study**.

A flow chart of clusters and individual participants through each phase of the study is shown in figure 1.

Of the 2,827 subjects considered for this analysis, 50% were men and the mean age was 42.8 (Standard Deviation = 13.6).

At baseline, men were significantly older and consumed more alcohol than women. Men also started to smoke earlier, consumed more tobacco and had higher nicotine dependence than women. There were more men than women in the preparation and action stages, and men were more confident and prepared to quit than women (Table 1). When the analysis was stratified by study group (Table 2), differences were found between men and women in both groups at baseline. The difference in the Fagerström test scores was statistically significant in the control group (p = 0.004), and stage, confidence in quitting and readiness to quit were only statistically significant in the intervention group (p = 0.010, p < 0.001, and p = 0.048 respectively). The key changes between genders among current smokers interviewed one year after their inclusion in the study were differences in confidence in quitting, which were observed in the control group (p = 0.044) but not in the intervention group (p = 0.206).

**Table 1:** Distribution of socio-demographic information, lifestyle information and tobacco consumption characteristics according to gender at baseline.

	Total	Men (%)	Women (%)	p-value
Sample size (n)	2827	1413 (50)	1414 (50)	
group				
control	1345	699 (47.3)	676 (47.8)	
intervention	1482	744 (52.7)	738 (52.2)	0.806
age (years), mean (SD)	42.8 (13.6)	45.9 (14.5)	39.7 (11.8)	**<0.001**
social class				
I-II	491	236 (17.4)	255 (20.1)	
III-V	2131	1120 (82.6)	1011 (79.9)	0.073
alcohol intake*, mean (SD)	6.0 (10.3)	9.2 (12.7)	2.8 (5.9)	**<0.001**
physical exercise				
never	1569	760 (54.3)	809 (57.9)	
≥ 1 per week	1228	639 (45.7)	589 (42.1)	0.059
age at smoking initation, mean (SD)	16.7 (4.5)	16.0 (3.9)	17.5 (5.0)	**<0.001**
n° cig/day, mean (SD)	20.4 (10.8)	22.1 (11.5)	18.7 (9.8)	**<0.001**
Fagerström test scores, mean (SD)	4.5 (2.5)	4.6 (2.5)	4.3 (2.5)	**0.002**
Richmond test scores, mean (SD)	6.3 (2.7)	6.3 (2.7)	6.2 (2.7)	0.191
smoker partner				
yes	1183	499 (41.2)	684 (59.6)	
no	1174	711 (58.8)	463 (40.4)	**<0.001**
smoker friends				
yes	2477	1242 (89.7)	1235 (89.4)	
no	288	142 (10.3)	146 (10.6)	0.788
stage				
pre-contemplation	643	326 (23.4)	317 (22.9)	
contemplation	1455	688 (49.5)	767 (55.4)	
preparation	554	302 (21.7)	252 (18.2)	
action	123	75 (5.4)	48 (3.5)	**0.002**
No. of previous attempts to quit lasting ≥ 24 hrs				
0	812	356 (25.6)	456 (32.8)	
1	511	254 (18.2)	257 (18.5)	
2	403	186 (13.4)	217 (15.6)	
3	298	153 (11.0)	145 (10.4)	
4	170	87 (6.3)	83 (6.0)	
5	588	356 (25.5)	232 (16.7)	**<0.001**
reduction no. cig over last month				
yes	1453	722 (52.2)	731 (53.2)	
no	1303	660 (47.8)	643 (46.8)	0.614
importance of quitting smoking (rank 0-10), mean (SD)	7.8 (2.6)	7.8 (2.6)	7.9 (2.5)	0.169
confidence in quitting smoking (rank 0-10), mean (SD)	4.9 (3.1)	5.1 (3.1)	4.6 (3.2)	**<0.001**
readiness to quit smoking (rank 0-10), mean (SD)	5.1 (3.1)	5.4 (3.1)	4.8 (3.1)	**<0.001**
tobacco-related disease				
yes	1171	692 (52.0)	479 (36.2)	
no	1481	638 (48.0)	843 (63.8)	**<0.001**

ISTAPS study.

SD: Standard Deviation

* measure: standard unit of alcohol per week (1 unit = 10 gr) (no drinkers included)

**Table 2:** Differences between genders in control and intervention groups at baseline and at one-year follow-up.

Baseline (restricted to individuals from whom information is available at one year)								
	**Control n (%) = 703 (48.2)**				**Intervention n (%) = 754 (51.8)**			
	**total**	**men**	**women**	**p-value**	**total**	**men**	**women**	**p-value**
no. cig/day, mean (SD)	20.2 (10.6)	22.1 (11.6)	18.7 (9.4)	**<0.001**	20.7 (10.5)	22.1 (10.9)	19.5 (10.0)	**0.001**
Fagerström test scores, mean (SD)	4.5 (2.5)	4.8 (2.6)	4.3 (2.5)	**0.004**	4.7 (2.5)	4.6 (2.5)	4.7 (2.5)	0.609
Richmond test scores, mean (SD)	6.0 (2.7)	6.1 (2.7)	5.9 (2.8)	0.198	6.2 (2.8)	6.2 (2.8)	6.3 (2.7)	0.755
stage								
pre-contemplation	176	73 (3.3)	103 (27.3)		178	92 (27.6)	86 (21.0)	
contemplation	376	166 (53.0)	210 (55.7)		397	156 (46.8)	241 (58.9)	
preparation	121	64 (20.4)	57 (15.1)		139	69 (0.7)	70 (17.1)	
action	17	10 (3.2)	7 (1.9)	0.151	28	16 (4.8)	12 (2.9)	**0.010**
reduction no. cig over last month								
yes	365	174 (55.1)	191 (50.1)		370	153 (47.2)	217 (53.8)	
no	332	142 (44.9)	190 (49.9)	0.194	357	171 (52.8)	186 (46.2)	0.076
importance of quitting smoking, mean (SD)	7.7 (2.6)	7.6 (2.5)	7.8 (2.6)	0.181	7.7 (2.6)	7.6 (2.8)	7.9 (2.5)	0.195
confidence in quitting smoking, mean (SD)	4.4 (3.1)	4.5 (3.0)	4.3 (3.1)	0.474	4.8 (3.1)	5.2 (3.0)	4.4 (3.1)	**<0.001**
readiness to quit smoking, mean (SD)	4.6 (3.0)	4.8 (2.9)	4.5 (3.1)	0.146	4.9 (3.1)	5.2 (3.2)	4.7 (3.0)	**0.048**

**One-year follow-up**								

	**Control n (%) = 703 (48.2)**				**Intervention n (%) = 754 (51.8)**			
	**total**	**men**	**women**	**p-value**	**total**	**men**	**women**	**p-value**
no. cig/day, mean (SD)	16.9 (9.9)	18.3 (11.2)	15.8 (8.5)	**0.009**	15.9 (9.5)	17.0 (10.5)	15.1 (8.6)	**0.013**
Fagerström test scores, mean (SD)	--	--	--		--	--	--	
Richmond test scores, mean (SD)	--	--	--		--	--	--	
stage								
pre-contemplation	226	97 (30.7)	129 (33.8)		219	97 (28.8)	122 (29.3)	
contemplation	252	112 835.4)	140 (36.6)		307	129 (38.3)	178 (42.8)	
preparation	220	107 (33.9)	113 (29.6)	0.454	227	111 (32.9)	116 (27.9)	0.283
reduction no. cig over last month								
yes	348	157 (49.7)	191 (50.5)		382	168 (50.3)	214 (51.8)	
no	346	159 (50.3)	187 (49.5)	0.824	365	166 (49.7)	199 (48.2)	0.680
importance of quitting smoking, mean (SD)	8.0 (2.5)	8.0 (2.4)	8.1 (2.5)	0.524	8.0 (2.4)	7.9 (2.4)	8.0 (2.4)	0.749
confidence in quitting smoking, mean (SD)	4.3 (3.1)	4.5 (3.0)	4.1 (3.1)	**0.044**	4.2 (2.9)	4.4 (2.9)	4.1 (2.9)	0.206
readiness to quit smoking, mean (SD)	4.3 (2.9)	4.7 (2.9)	4.0 (3.0)	**0.002**	4.4 (2.9)	4.8 (3.0)	4.1 (2.8)	**0.001**

Only smokers. ISTAPS study.

SD: Standard Deviation

The variables associated with number of cigarettes per day, importance, confidence and readiness to quit of smokers at the one-year follow-up showed differences between men and women (Table 3). The most important difference is that physical exercise reduces significantly the number of cigarettes consumed daily in women. Differences between men and women for the remaining predictor variables are of lesser magnitude. Baseline values for each variable considered are highly significant predictors.

**Table 3:** Independent predictors of no. cig/day, importance of quitting, confidence in quitting and readiness to quit at one-year follow-up.

Men one-year follow-up				
	**N°cig/day**	**importance of quitting**	**Confidence in quitting**	**Readiness to quit**

	**Coefficient** **(robust SE^†^)**	**Coefficient** **(robust SE^†^)**	**Coefficient** **(robust SE^†^)**	**Coefficient** **(robust SE^†^)**

no. cig/day	0.5 (0.05)***			
Fagerström test scores	0.7 (0.19)***		-0.3 (0.05)***	-0.2 (0.05)**
Richmond test scores		0.2 (0.05)***		
importance of quitting		0.2 (0.04)***		
confidence in quitting			0.3 (0.04)***	0.2 (0.05)**
readiness to quit	-0.4 (0.12)***	-0.1 (0.04)*		0.1 (0.05)**

**Women** **one-year follow-up**				

	**N°cig/day**^‡^	**Importance of quitting**	**Confidence in quitting**	**Readiness to quit**

	**Coefficient** **(SE)**	**Coefficient** **(robust SE^†^)**	**Coefficient** **(robust SE^†^)**	**Coefficient** **(robust SE^†^)**

physical exercise > = 1 per week [ref.never]	-1.8 (0.49)***			
no. cig/day	0.4 (0.03)***			
Fagerström test scores	0.4 (0.13)***		-0.2 (0.04)***	-0.2 (0.04)***
Richmond test scores		0.1 (0.04)***		
Importance of quitting		0.3 (0.04)***		
confidence in quitting	-0.2 (0.10)*	-0.1 (0.03)***	0.3 (0.04)***	
readiness to quit	-0.2 (0.10)*			0.2 (0.04)***

Linear regression model. ISTAPS study

SE: standard error

Predictors variables were measured at baseline

Final models were adjusted only for significant variables.

^* ^p ≤ 0.05 ^** ^p ≤ 0.01 ^***^p ≤ 0.001

^† ^The standard errors were adjusted for the cluster effect of the Basic Care Unit

^‡ ^Linear mixed-effects model

The abstinence rate (point prevalence) at the one-year follow-up was 18.2% in the intervention group versus 13.8% in the control group (p = 0.002). By gender, the abstinence rate (point prevalence) was 17.2% for men and 15% for women (p = 0.118).

At the one-year follow-up, the six-month continuous abstinence rate was 11.3% and 6.3% in the intervention and control group respectively (p < 0.001). By gender, the rates were 9.4% for men and 8.5% for women (p = 0.400).

The logistic mixed-effects model showed that women had non-significant lower odds of being an ex-smoker than men after the analysis was adjusted for study group, age, alcohol intake, number of cigarettes per day, Fagerström test scores, Richmond test scores, confidence in quitting and readiness to quit smoking at baseline (adjusted OR = 0.9, 95% CI = 0.7-1.2). Statistically significant variability was found between BCUs (variance = 0.2, 95% CI = 0.1-0.7) (Table 4).

**Table 4:** Predictors of smoking cessation (ex-smokers versus smokers).

	Unadjusted OR (CI 95%)	Adjusted OR (CI 95%)	p-value
*Fixed effects*			
gender			
men	1	1	
women	0.9 (0.7-1.2)	0.9 (0.7-1.2)	0.696
group			
control	**1**	**1**	
intervention	**1.9 (1.4-2.6)**	**1.7 (1.2-2.4)**	**0.001**
age (years)	1.0 (1.0-1.0)	1.0 (1.0-1.0)	**0.011**
alcohol intake	0.9 (0.9-1.0)	0.9 (0.9-1.0)	**0.003**
no. cig/day	1.0 (1.0-1.0)	1.0 (1.0-1.0)	**0.003**
Fagerström test scores	0.9 (0.9-1.0)	**0.9 (0.8-0.9)**	**<0.001**
Richmond test scores	**1.2 (1.1-1.2)**	1.1 (1.0-1.2)	**0.002**
confidence in quitting smoking	**1.1 (1.1-1.2)**	1.1 (1.0-1.1)	0.091
readiness to quit smoking	**1.2 (1.1-1.2)**	1.1 (1.0-1.1)	0.207

* Random effects parameter*	* Estimate (95%CI)*		
Variance (BCU) (2 level)	**0.2 (0.1-0.7)**		

Logistic mixed-effects model. ISTAPS study.

Final model were adjusted for significant and/or confounder variables

Likelihood ratio test vs logistic regression p = 0.0137

## Discussion

This study focused on the role of gender as a potential predictor of smoking cessation after controlling for potential confounders such as age and amount smoked, among other variables.

This study, which was carried out in a large number of sites, was aimed primarily at assessing the role of gender rather than focusing on the effect of the intervention, as tobacco consumption in Spain is increasing among women.

Therefore, gender differences were consider in socio-demographic and lifestyle factors, as well as in the patterns of tobacco use. Almost all p values between genders in Table 1 were statistically significant. Part of this effect was due to the large sample size of our study; however, this effect was also partly due to the existence of a gender pattern in socio-demographic and socio-economic variables, as described in the Spanish National Health Survey conducted in 2006 [4].

Females had different habits than males in relation to tobacco consumption; for example, men started to smoke at a younger age, and their consumption of cigarettes per day was higher than in women, as has been reported in other studies [9,22,23]. Men also had greater nicotine dependence; as many studies have demonstrated, nicotine addiction is the main barrier to cessation [1,24]. Although women seem to be more conscious of their health, the six-month continuous abstinence rate was slightly higher in men than in women, as reported previously [25,26]. However, this difference observed between genders in our study was not statistically significant. In the univariate analysis, men were more confident and ready to quit smoking than women [9]. In this study, male smokers were older than female smokers, which reflects the actual situation in the Spanish population [4]. The difference observed in the levels of confidence and readiness to quit between genders could stem from the fact that men were older than women and that there is a clear tendency to quit smoking in older age groups, as reported by a study conducted in Britain [27].

The existing evidence regarding gender and smoking cessation interventions has so far been diverse. Studies by Bohadana et al., Pogun et al. and others authors show that the male gender, among other factors, is usually considered to be a predictor of a successful outcome in smoking cessation [25,28-30]. Women seem to resort to smoking to confront difficult situations that cause anxiety or depression. Furthermore, women who are concerned about post-cessation weight gain are less motivated to quit smoking [31,32]. These factors may contribute to the observation that women have poorer smoking cessation outcomes. Another reason that could explain why women are not as successful in smoking cessation is the poorer effect of nicotine replacement therapies in women. A number of studies show that more men than women quit smoking after using nicotine replacement therapies (NRT), indicating that NRTs are less effective in female smokers [33]. However, it has been reported that women and men benefited equally from treatment with Bupropion [34]. It should be emphasized that NRTs or bupropion cannot be assessed for individual effectiveness on smoking cessation in our study, since these treatments were considered to be a part of the intervention.

However, in a group of studies, no gender differences were found in the abstinence rates [22,35]. When gender was adjusted for potential confounders, no gender differences in smoking cessation were found as we have observe in our study [23,36]. Using evidence from studies that vary in design, sample characteristics, and intensity of the interventions studied, researchers to date have not found consistent gender-specific differences in the effectiveness of intervention programs for tobacco cessation.

Other studies have reported that females are more likely to have achieved a six-month abstinence [35], perhaps because the higher percentage of female smokers responding to follow-up [37]. Nevertheless, many studies have not reported cessation results by gender [36].

There is limited information related to importance, confidence and readiness to quit smoking and gender differences in the literature. Specifically, there is no literature regarding the predictors that could increase the importance of quitting, self-confidence in quitting and readiness to quit at one-year follow-up. Some gender differences were observed in these variables at one-year follow-up in our study, but further research is warranted to support these results.

### Study strengths and limitations

In this study, which included several important factors associated with smoking cessation, the effect of gender was adjusted for confounding factors. Moreover, our study took into account the design effect in the calculation of the sample size, as well as the clustering of individuals within BCUs in the analysis.

Another strength of the study is the large number of participants. Furthermore, the study has great external validity since it was undertaken in several regions of Spain. All of the procedures were standardized, and all participating sites were coordinated by the same coordinating center. In addition, the professionals of both study groups received training before beginning the study. E-mails were sent each week, and meetings were held periodically to ensure continuity and quality of the study procedures.

Also, this study is one of the few to analyze gender when transtheoretical measures are included as predictor variables [9].

Some limitations of this study should be noted in interpreting the findings. One of the limitations was the recall bias related to dates. Patients may not have correctly remembered dates that were used in data collection, such as when they quit smoking. However, recall bias is normal in studies that use self-reported data.

The fact that patients were asked if they wanted to participate in the study could have had a therapeutic effect that improved their willingness to quit smoking. However, this therapeutic effect is present in both the control and the intervention groups, and in both genders.

There were only two time points of data collection, with a one-year interval [24]. Therefore, we do not have complete information about process or attitudes in the middle of the study period.

An important limitation in this study is the percentage of subjects lost to follow-up. Because the percentages of subjects lost to follow-up are similar in both groups (32.4% in the intervention group and 34.6% in the control group) and a penalizing data input method was used (subjects lost to follow-up were considered to be smokers), we do not believe this to represent a bias for estimating the relative effect of the intervention. However, this does result in a bias for estimating the absolute effect of both the intervention and the standard care administered in the control group. Indeed, if the analysis was performed only with subjects who were followed, the point abstinence rate at one year would be 24.5% in the intervention group and 20.1% in the control group. The actual abstinence rate is believed to lie somewhere between that value and the more conservative one which was reported in the Results section above. Additionally, we believe that this percentage of dropouts may result from the complexity of the study, which was conducted at 82 healthcare centers in several regions of Spain and was incorporated to the routine of the visits. According to the CONSORT guidelines, all cases should be assessed as originally allocated to their study group under an intention-to-treat approach, and data for missing subjects should be input using the most suitable method, especially when there is a similar percentage of dropouts in both groups. In any case, no input method is free of bias.

One of the theoretical foundations for the study was the stages of change model. It is unclear how much time is needed to change a particular behavior, since not many differences were found in the stages of change between the baseline interview and the one-year interview.

## Conclusions

We can conclude that gender does not appear to be a predictor of smoking cessation in individuals presenting at Primary Care Centers in Spain when the effect is adjusted for potential confounders. Further educational and public health campaigns are needed to encourage the use of pharmacological and non-pharmacological interventions in both genders. The study of characteristics associated with tobacco abstinence is important for assisting health care providers in tailoring interventions and for improving the quality of clinical services provided [37].

This study serves as a basis for the intervention used today in many Spanish Primary Care Centers, and some of the assessments proposed have been included in the clinical practice.

## Conflict of interest

The authors declare that they have no competing interests.

## Authors' contributions

DP took responsibility for the cleanup and maintenance of the database as well as processing the manuscript. CC, CF, TC, MT, LC and CM participated in the design of the study and helped to draft the manuscript. TR performed part of the statistical analysis. All authors read and approved the manuscript.

## ISTAPS study group investigators (IAPP Network Node)

- **Director Team**: IDIAP J Gol (principal investigator: Carmen Cabezas); **co-investigators**: Carlos Martin, Silvia Granollers, Concepció Morera, Josep lluis Ballvé, Elvira Zarza, Margarida Borràs, Antoni Serra, Diana Puente, Teresa Rodriguez-Blanco, Joan Lozano. **Research fellows**: Xavier González, Valeria Pacheco, Xavier Blancafort, Mamta Advani

- **Andalucia-Al Andalus**: Isabel Fernandez Fernandez (Network Node Coordinator) Transito Cebrian and Inmaculada Romero (Local Research Supervisors), Beatriz López, César J. Costa, Mª Isabel Villafuerte, Vicente Rodríguez Pappalard, Antonia Aguilera, José Manuel Santos, Filomena Ballester, Francisco J. García De La Corte, Emilio Márquez

- **Baleares- IB Salut**: Joan Llobera (Network Node Coordinator), Eugenia Carandell and Micaela Llull (Local Research Supervisors), Sebastià March (Research student), Llull, Irene López, Ana Badosa, Antonia Gual, Mercé Gomila, Miguel Angel Vicente, Lucia Moreno, Miquel Góngora, Tina Crespi, Irene Sempere, Adoración Viñals, Santiago Alegret, Carmen Marques, Francisca Bestard, Juana Rossinyol, Marian Llorente, Ana Uréndez

- **Catalunya - GIAP1 and 2**: Xavier González (Research student), Montserrat Grivé, Mercè Fortín, Francesc Balaguer, Eva Peguero, Aurora Garriga, Neus Fernández, Concepció López, Carme Roca, Neus Profitós, Antoni Vives, Xavier Monteverde, Marta Constanza, Gemma Fanlo, Jaume Doménech, Francesca Ruiz, Joan Lozano, Carme Batalla, Begoña González, Yolanda Ortega, Nuria Martín, Carles López, Josep Puig, Margarida Puigvert, Fernando Montesinos, Ramon Casas, Inmaculada Vázquez, Juan Antonio Sabio, Amparo Gaitano, Fernando Ferrer, Mª Jesús Avila, Jacint Caula, Ricard Tell, Leonor Bosch, Juan José Mascort, Marta Sanavia, Rosario Aguado, Santiago Pérez, Guadalupe Ortega, Rafel Ramos, Miquel Quesada, Carme Jiménez, Raquel Burón, Lourdes Crespo, Elisabet Rayó, Judit Vila, Enric Simó, Meritxell Coll

- **Castilla la Mancha**: Vicente Martínez Vizcaíno (Network Node Coordinator), Mª Dolores Ruíz (Local Research Supervisors), Blanca Notario (Research student), Angel García, Pilar Figueroa, Tomás Carrasco, Carmen Sáiz, Adoración Romero, Miguel Angel Laparra, Sagrario Saiz, Gerardo Bollo, Jesús Buendía, Nieves Valero, Esmeralda García, Rosa López, Moisés Molina, Mª Luz Ladrón, Mª Dolores Ruiz, Cristina Lafuente, Yolanda Jarabo, Fortunato Muelas, Fernando Madero, Mª Jesús Cuesta

- **Castilla León**: Carmen Fernández Alonso (Network Node Coordinator), Miguel Torrecilla and Aventina de la Cal (Local Research Supervisors), Doradia García, Ana Isabel Miranda, Manuel Gómez, Mar Gonzales, Severina Martín, Miguel Torrecilla, Elisa León, Paz Muriel, Susana Domínguez, Dolores Plaza, Raquel Ruano, Pilar Moreno, Carmen Castaño, Leonor Collazos, Ana Teresa Asensio, Patricia Hernández, Aventina De La Cal, Cristina Rodríguez, Teresa Postigo, Yolanda Valpuesta, Felix Pastor

- **Galicia - GIAP**: Pilar Gayoso Díaz (Network Node Coordinator), Felisa Domínguez and Xulio Castañal (Local Research Supervisors), Begoña Dominguez, Luciano Casarieg, Isabel Cortés, Ana Carvajal, Maria Del Pilar Pintos, Manuel Liñares, Joaquina Campo, Antonio Roberto Barca, Antonio González, Milagros Núñez, Carmen Maria Riva, Mª Carmen Zoila, Rosa Saenz, Concepción Meira, Manuel Rodríguez, Mercedes Rodríguez, Alberto Del Alam, Angeles Rodríguez, Gloria Antuña López

- **Madrid - AREA 11**: Tomás Gómez Gascón (Network Node Coordinator), Julia Domínguez (Local Research Supervisors), Alicia Ramos [Research student], Soledad Martín, Maria Escutia, Asunción Prieto, Elena Méndez-Bonito, Mercedes Parrilla, Mercedes Medina, José Nemesio Villaroel, Mercedes Fuentes, Isabel Pérez, Ramón Cuenca, Rosario Novo, Pablo Quintana, Fernando Villasante, Francisco De Lucas, Cesar Jurad, Begoña Manzanero, Javier Amador, Isabel Fernández, Silvia Ayala, Carmina Batlle, Olga Rupérez, Benito Del Pino

- **Madrid-Prev**: Francisco Camarelles (Network Node Coordinador and Local Supervisor), Enrique Carrillo (Research student), Blanca Sanz, Braulia Calvo, Maria Cortés Catalan, Maribel Morente, Carmen Azpeitia, Dolores Rodero, Isabel Edo, Mª Amor Fraile, Beatriz Becerril, Araceli Garrido, Tomasa Montes, Victoria Aguirre, Elena Berezibar, Javier Castellanos, Mª Teresa Fernández, Esperanza Calvo, Encarnación Vega, Milagros Velázquez, Mª Carmen Belinchón

- **Extremadura**: Francisco Buitrago (Network Node Coordinator and Local Supervisor), Lourdes Cañon (Research student), Teresa Nieto, Timotea Garrote, Mª José Gamero, Elisabeth Navarro, Manuel Espigares, Mª Carmen Paniagua, Leoncio Rodríguez, Mª Carmen Risco, Tomás Vega, Jose A. Morales, Manuel Comellas, Espinosa García

- **Andalucia-COGRAMA**: Francisca Leiva (Network Node Coordinator), Marieta Catalán and Rodrigo Mayo (Local Research Supervisors), Carmen Posadas, Antonio Hernández, Josefa Díaz, Rafael Garófano, Antonia Navas, Amapola Herrera, Juan José Herrera, Elena Requejo, Javier Jiménez, José Leiva, Pilar Aguirre, Rafael Angel Maqueda, Francisca Santana, Maria Catalán, Rosa Olmedo, Mercedes Fernández, Francisco Escalante, Manuel Martínez, Mariano Ropero, José Eloy Téllez, José Antonio Ruiz Pretel, Alejandro Flores Barranco, Belén Martín Gálvez, José Enrique Díaz Rodríguez, Teresa Parejo, José Venegas, Inmaculada Peláez, Blas Mayor, Jesús Beltrán, Juan Carlos Rico, Fuensanta Lozano, Antonio Cuadra, Teresa Cantos

- **Cantabria**: Ana Sobrino (Network Node Coordinator), Esther Quintanal (Local Research Supervisors), Liébana Piedra and Manuel Tazón (Research student), Susana Rodríguez, Elena Basabe, Ana Solórzano, Pilar De La Puebla, Rocio Pérez, Luisa Alonso, Jose Antonio Rio, Esther Quintanal, Pilar García, Pilar Villanueva, Jose Gabriel Pinedo, Enriqueta García, Yolanda Cuerno, Mercedes Losilla, Eduardo Olavarri, Fernando Quijano, Jesús Arnaiz, Mª José Iribarren, Nicanor Valle, Jesús Castillo, Santiago Raba, Ana Lavín

- **Aragón**: Rosa Magallón (Network Node Coordinator), Antonio Castillón and Sara Fanlo (Local Research Supervisors), Bárbara Oliván (Research student), Daniel Escribano, Antonio Castillón, Lourdes Clemente, José Bernués, Dolores Idañez, Mª Angeles Pardo, Virginia López, Teofilo Lorente, Beatriz Solans, Francisco Domínguez, Virtudes Peregrina

- **Valencia**: Carles Sanchos (Network Node Coordinator and Local Supervisor), José Vicente Galán and Ruth Victorio (Research student), Mª Reyes Falcó, Mª Del Carmen Tarazona, Miquel Morera, Miguel Prosper, Ricardo García, Mª José Paredes, Vicenta Beti, Albert Pedro Salazar, Francisco Perís, Maria Isabel Luz Andrés, Lluis Monedero, Rosa Mª Albelda, Agustín Andrés, Rosana Espinosa, María García, Inmaculada Muñoz, Juan Grau, Javier Domingo, Mª Vicenta Ferrandis, Mª Del Carmen Serra, Mª Dolores Sanchís, Francisco Lluch, Dolores Mahique, José Manuel Soler, Clara Serra

## Pre-publication history

The pre-publication history for this paper can be accessed here:

http://www.biomedcentral.com/1471-2458/11/369/prepub

## References

[B1] U.S.Department of Health and Human Services The Health Consequences of Smoking:nicotine addiction. A Report of The Surgeon General DHHS Publication No. [CDC] 88-8406 2004 Public Health Service. United States

[B2] World Health Organization Global Health Risks: mortality and burden of disease attributable to selected major risks 2009 http://www.who.int/healthinfo/global_burden_disease/global_health_risks/en/index.html

[B3] Banegas JR Diez-Gañán L Bañuelos-Marco B Gonzalez-Enríquez J Villar-Álvarez F Martín-Moreno JM Córdoba-García R Pérez-Trullén A Jiménez-Ruiz C Mortalidad atribuible al consumo de tabaco en Espana en 2006 Med Clin [Barc] 2011 136 97 102 10.1016/j.medcli.2010.03.039 20980030

[B4] Ministerio de Sanidad y Consumo Español Encuesta Nacional de Salud 2006 http://www.msc.es

[B5] Comité Nacional para la Prevención del Tabaquismo Women and Smoking: A White Paper Addressing Smoking from a Gender Perspective 2009 Ministerio de Sanidad y Consumo Español

[B6] Payne S 'Smoke like a man, die like a man'?: a review of the relationship between gender, sex and lung cancer Soc Sci Med 2001 53 1067 1080 10.1016/S0277-9536(00)00402-0 11556776

[B7] Ferketich AK Khan Y Wewers ME Are physicians asking about tobacco use and assisting with cessation? Results from the 2001-2004 national ambulatory medical care survey [NAMCS] Prev Med 2006 43 472 476 10.1016/j.ypmed.2006.07.009 16920185

[B8] Doucet JM Velicer WF Laforge RG Demographic differences in support for smoking policy interventions Addict Behav 2007 32 148 157 10.1016/j.addbeh.2006.04.003 16814475

[B9] D'Angelo ME Reid RD Brown KS Pipe AL Gender differences in predictors for long-term smoking cessation following physician advice and nicotine replacement therapy Can J Public Health 2001 92 418 422 11799544 10.1007/BF03404531 PMC6979757

[B10] Prochaska JO DiClemente CC Velicer WF Ginpil S Norcross JC Predicting change in smoking status for self-changers Addict Behav 1985 10 395 406 10.1016/0306-4603(85)90036-X 4091072

[B11] Dotinga A Schrijvers CT Voorham AJ Mackenbach JP Correlates of stages of change of smoking among inhabitants of deprived neighbourhoods Eur J Public Health 2005 15 152 159 10.1093/eurpub/cki112 15941760

[B12] Cabezas C Martin C Granollers S Morera C Ballve JL Zarza E Blade J Borras M Serra A Puente D Effectiveness of a stepped primary care smoking cessation intervention [ISTAPS study]: design of a cluster randomised trial BMC Public Health 2009 9 48 19193233 10.1186/1471-2458-9-48 PMC2666720

[B13] Hughes JR Keely JP Niaura RS Ossip-Klein DJ Richmond RL Swan GE Measures of abstinence in clinical trials: issues and recommendations Nicotine Tob Res 2003 5 13 25 12745503

[B14] Cabezas C Martín C Ballve JL Blade J Borras M Granollers S Morera C Detecció i tractament del consum de tabac. Editat pel Institut Català de la Salut.Barcelona núm 14 Guies de pràctica clínica 200917076930

[B15] Heatherton TF Kozlowski LT Frecker RC Fagerstrom KO The Fagerstrom Test for Nicotine Dependence: a revision of the Fagerstrom Tolerance Questionnaire Br J Addict 1991 86 1119 1127 10.1111/j.1360-0443.1991.tb01879.x 1932883

[B16] Richmond RL Kehoe LA Webster IW Multivariate models for predicting abstention following intervention to stop smoking by general practitioners Addiction 1993 88 1127 1135 10.1111/j.1360-0443.1993.tb02132.x 8401167

[B17] Rollnick S Butler CC Stott N Helping smokers make decisions: the enhancement of brief intervention for general medical practice Patient Educ Couns 1997 31 191 203 10.1016/S0738-3991(97)01004-5 9277242

[B18] National Centre for Social Research British Social Attitudes. Perspectives on a changing society: British Registrar General's Social Class [Rep. No. 23] 2006

[B19] Barnes SA Larsen MD Schroeder D Hanson A Decker PA Missing data assumptions and methods in a smoking cessation study Addiction 2010 105 431 437 10.1111/j.1360-0443.2009.02809.x 20402986

[B20] Eldridge SM Ashby D Feder GS Rudnicka AR Ukoumunne OC Lessons for cluster randomized trials in the twenty-first century: a systematic review of trials in primary care Clin Trials 2004 1 80 90 10.1191/1740774504cn006rr 16281464

[B21] Raudenbush SW Bryk AS Hierarchical linear models: applications and data analysis methods 2002 2 Sage London

[B22] Gritz ER Thompson B Emmons K Ockene JK McLerran DF Nielsen IR Gender differences among smokers and quitters in the Working Well Trial Prev Med 1998 27 553 561 10.1006/pmed.1998.0325 9672949

[B23] Croghan IT Ebbert JO Hurt RD Hays JT Dale LC Warner N Schroeder DR Gender differences among smokers receiving interventions for tobacco dependence in a medical setting Addict Behav 2009 34 61 67 10.1016/j.addbeh.2008.08.010 18814974

[B24] Zhu SH Sun J Billings SC Choi WS Malarcher A Predictors of smoking cessation in U.S. adolescents Am J Prev Med 1999 16 202 207 10.1016/S0749-3797(98)00157-3 10198659

[B25] Bohadana A Nilsson F Rasmussen T Martinet Y Gender differences in quit rates following smoking cessation with combination nicotine therapy: influence of baseline smoking behavior Nicotine Tob Res 2003 5 111 116 10.1080/1462220021000060482 12745512

[B26] Carlson LE Goodey E Bennett MH Taenzer P Koopmans J The addition of social support to a community-based large-group behavioral smoking cessation intervention: improved cessation rates and gender differences Addict Behav 2002 27 547 559 10.1016/S0306-4603(01)00192-7 12188591

[B27] Royal College of Physicians: Nicotine Addiction in Britain A report of the Tobacco Advisory Group of the Royal College of Physicians 2000 Royal College of Physicians of London. London

[B28] Pogun S Yararbas G Sex differences in nicotine action Handb Exp Pharmacol 2009 192 261 291 10.1007/978-3-540-69248-5_10 19184653

[B29] Aubin HJ Lebargy F Berlin I Bidaut-Mazel C Chemali-Hudry J Lagrue G Efficacy of bupropion and predictors of successful outcome in a sample of French smokers: a randomized placebo-controlled trial Addiction 2004 99 1206 1218 10.1111/j.1360-0443.2004.00814.x 15317642

[B30] Osler M Prescott E Godtfredsen N Hein HO Schnohr P Gender and determinants of smoking cessation: a longitudinal study Prev Med 1999 29 57 62 10.1006/pmed.1999.0510 10419801

[B31] Weekley CK Klesges RC Reylea G Smoking as a weight-control strategy and its relationship to smoking status Addict Behav 1992 17 259 271 10.1016/0306-4603(92)90031-P 1636473

[B32] Levin RF Stout JM Singleton JK Londrigan M Feldman HR McMillan E Gender's effect on the efficacy of smoking cessation interventions Nurs Times 2004 100 32 34 14999829

[B33] Cepeda-Benito A Reynoso JT Erath S Meta-analysis of the efficacy of nicotine replacement therapy for smoking cessation: differences between men and women J Consult Clin Psychol 2004 72 712 722 15301656 10.1037/0022-006X.72.4.712

[B34] Scharf D Shiffman S Are there gender differences in smoking cessation, with and without bupropion? Pooled- and meta-analyses of clinical trials of Bupropion SR Addiction 2004 99 1462 1469 10.1111/j.1360-0443.2004.00845.x 15500599

[B35] Chatkin JM Abreu CM Blanco DC Tonietto R Scaglia N Wagner MB Fritscher CC No gender difference in effectiveness of smoking cessation treatment in a Brazilian real-life setting Int J Tuberc Lung Dis 2006 10 499 503 16704030

[B36] U.S.Department of Health and Human Service Women and Smoking: a report of the Surgeon General. Report No.: MMWR 2002; 51 [No.RR-12]:1-30 2002 Public Health Service. United States 12222832

[B37] Ferguson JA Patten CA Schroeder DR Offord KP Eberman KM Hurt RD Predictors of 6-month tobacco abstinence among 1224 cigarette smokers treated for nicotine dependence Addict Behav 2003 28 1203 1218 10.1016/S0306-4603(02)00260-5 12915164

